# Microbiome-gut-brain axis in Nutritional Psychiatry

**DOI:** 10.1192/j.eurpsy.2023.35

**Published:** 2023-07-19

**Authors:** H. Schellekens

**Affiliations:** ^1^Department of Anatomy & Neuroscience, University College Cork; ^2^APC Microbiome Ireland, Cork, Ireland

## Abstract

**Abstract:**

The last decade has seen an enormous expansion on our knowledge of the human gut microbiome and its importance for human physiology. The gastrointestinal inhabitants have taken centre stage as regulators of the bidirectional gut to brain communication. The gut microbiota is not only critical for metabolism, glucose homeostasis and body composition, but increasing evidence is demonstrating the significant effects of the gut microbiota on mood and mental wellbeing and its role in the development of affective disorders, such as anxiety and depression, and other neuropsychiatric conditions. Studies in the field of nutritional neuroscience and nutritional psychiatry are now increasingly including the gut microbiota as a key factor mediating the impact of diet on central nervous system function. Accumulating evidence from cell-based in vitro studies, animal models and preclinical intervention studies are linking the gut microbiota to the effects of diet on brain function, but the precise mechanism are still not fully understood and studies have had limited translation to human intervention studies. Overall, the increase in our understanding of interconnectedness of the gastrointestinal microbiota of human health and disease, has led to a strong focus on the development of microbiota-targeted strategies to influence all host physiological responses, including those that can modulate central nervous system function. In this talk, I will provide an overview of the most recent advances in the nutritional psychiatry–microbiome field, highlighting significant opportunities for future research.

**Disclosure of Interest:**

None Declared

